# User-based representation of time-resolved multimodal public transportation networks

**DOI:** 10.1098/rsos.160156

**Published:** 2016-07-13

**Authors:** Laura Alessandretti, Márton Karsai, Laetitia Gauvin

**Affiliations:** 1 Université de Lyon, ENS de Lyon, LIP, INRIA-CNRS-UMR 5668, IXXI, 69364 Lyon, France; 2 Data Science Lab, ISI Foundation, Turin, Italy; 3 Department of Mathematics, City University London, London EC1V 0HB, UK

**Keywords:** public transportation, multimodal networks, human dynamics

## Abstract

Multimodal transportation systems, with several coexisting services like bus, tram and metro, can be represented as time-resolved multilayer networks where the different transportation modes connecting the same set of nodes are associated with distinct network layers. Their quantitative description became possible recently due to openly accessible datasets describing the geo-localized transportation dynamics of large urban areas. Advancements call for novel analytics, which combines earlier established methods and exploits the inherent complexity of the data. Here, we provide a novel user-based representation of public transportation systems, which combines representations, accounting for the presence of multiple lines and reducing the effect of spatial embeddedness, while considering the total travel time, its variability across the schedule, and taking into account the number of transfers necessary. After the adjustment of earlier techniques to the novel representation framework, we analyse the public transportation systems of several French municipal areas and identify hidden patterns of privileged connections. Furthermore, we study their efficiency as compared to the commuting flow. The proposed representation could help to enhance resilience of local transportation systems to provide better design policies for future developments.

## 1. Introduction

Urban transportation systems interweave our everyday life and although their construction is based on conscious design they appear with complex structural and dynamical features [1]. They build up from different transportation means, which connect places in a geographical space. Their most straightforward description is given by networks [2,3] where stations are identified as nodes, and links are the transportation connections between them. Based on this representation [4], considerable research efforts have been dedicated to address their sustainability [5], to optimize their efficiency [6,7], reliability [8–10] or even to estimate the risk they carry due to interdependency with other infrastructure networks in case of terrorist attacks [11].

All transportation networks share a few common features: (i) they are all embedded in space, setting constraints in their structural design, (ii) networks of different transportation means may coexist in the same space and (iii) they are all inherently temporally resolved. Such details of several transportation networks became available lately [12] through the collection of large open datasets describing complete multimodal transportation systems in cities, regions, countries and even internationally. These advancements were induced by novel data collection techniques including smart card data [13], automatic vehicle location (AVL) data [14] and mobile phone data from GSM providers [15]. On the top of these developments, the advent of a new common non-proprietary transit data format, the General Transit Feed Specification (GTFS), further amplified actual trends in urban policy propagating smart city programmes and real-time online user services. As of February 2016, 325 public transportation companies around the world have released official GTFS feeds [16], which are regularly modified by online communities that are adding extensions and optional fields to adapt to different transit services [17]. In transportation, the confluence of open data, GTFS, ubiquitous mobile computing, sensing and communication technologies, has allowed to study the efficiency and performance of public transportation systems under different perspectives [18–22]. As a consequence, GTFS data are now used for trip planning, ride-sharing, timetable creation, mobile data, visualization, accessibility and to provide real-time service informations.

These recent developments in data collection practices and in the corresponding fields of complex networks and human dynamics provided the opportunity to quantitatively study transportation systems using a data-driven approach. These studies showed that geographical constraints largely determine the structure and scaling of transportation networks [23–25], but for their better understanding one needs to consider the actual urban environment and development level [7,26,27]. At the same time, the emerging field of multilayer networks provided the methodology to consider their multimodal character [28,29]. In this representation, each layer corresponds to the network of a single means of transportation (bus, tram, train, etc.), which are defined on the same set of nodes (stations). This way they account for possible multiple links of different modes between the same stations [30]. This representation can be extended to capture the temporal nature of the system by using some aggregated information extracted from the transportation schedule [31] or, as a future challenge, by considering each time slot as a layer where journeys between stations are represented as temporal links [28,32].

Here, we build on these contemporary advancements and provide a novel representation, which combines multi-edge and P-space representations of transportation networks. The proposed scheme considers the system from the user’s point of view by incorporating the minimization of the total travel time, its variability across the schedule, and the number of transfers between lines. Our subsequent aim is to adjust earlier-defined characterization techniques to the proposed representation in order to help its analysis. We use the adjusted techniques (i) to identify patterns of privileged connections in the transportation network, which are not evidently present through their overall design, and (ii) to quantify their overall efficiency when compared with the commuting flow. We carried out our analysis using openly shared GTFS datasets describing extensive transportation networks of French municipalities, such as greater Paris, Toulouse, Nantes and Strasbourg.

As follows, first, we describe the actual time-resolved multilayer network representation and introduce our methodology incorporating travel routes and times to identify efficient transportation connections. Next, we apply a matrix factorization method to extract underlying connectivity patterns to analyse them from the commuter point of view, and quantify their overall efficiency. Finally, we conclude our results and discuss possible applications and future directions of research. Note that the implementation of the proposed methodology is openly accessible online (https://github.com/lalessan/user_basedPT).

## 2. Representation of public transportation networks

The proposed methodology integrates several sequential steps to detect origin–destination areas that are conveniently connected by public transportation with respect to user preferences. In the following description, first, we define a user-based representation of a public transportation (PT) system, which limits the effect of its spatial embeddedness, but accounts for its multilayer structure, and its temporal dimension. Next, we calculate the shortest time paths between stops by adapting a conventional algorithm [33] to the actual graph representation, and finally, we select preferred connections, taking into account the distance travelled over time.

### 2.1. User-based multi-edge P-space representation

**Figure 1.**
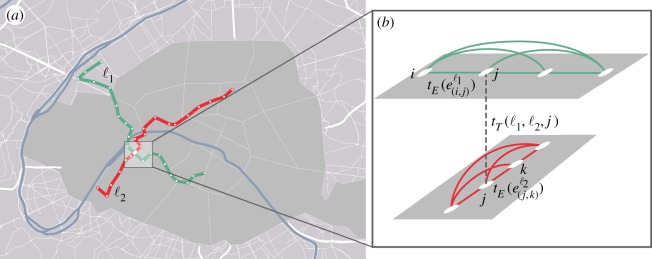
Illustration of the user-based multi-edge P-space representation. (*a*) Two geo-localized crossing PT lines ℓ_1_ and ℓ_2_ are shown on the map of central Paris. (*b*) Schematic of the P-space multi-edge representation for a section of the network: all pairs of nodes corresponding to stops on the same line are connected by edges with the same label.

Earlier studies revealed that the choice of users to select transportation means for commuting is mainly affected by the average travel time, and by the variability of the total travel time [34],^1^ [35],^2^ in addition to the number of transfers they need to do. Our principal goal here is to introduce a novel representation of PT networks, which incorporates the aforementioned aspects decisive for users (while neglecting other presumably less determinant factors such as travel cost or comfort), and which minimizes the effects due to the spatial embeddedness of the system. In order to do so, we combine a multi-edge [36] and a P-space representation of the transportation network [37–39] to describe the PT systems. The multi-edge representation accounts for the presence of several transportation lines in the same PT network by allowing the existence of multiple labelled edges within a single pair of nodes. On the other hand, the P-space representation takes into account that transfers between lines is time-consuming and may not be convenient for the user; also, it considers connections between stops located at large distance thus it reduces the effect of the geographical distances. The combination of these two representations constitutes an ideal framework to investigate complex features of PT systems from the user perspective. A schematic example of this representation is displayed in figure 1*a*, showing two crossing PT lines, and in figure 1*b*, we illustrate the corresponding P-space multi-edge representation. Two stops *i* (respectively, *k*) and *j* on the same line ℓ_1_ (respectively, ℓ_2_) are linked through the edge $e_{\left(i,j\right)}^{\ell_{1}}$ (respectively, $e_{\left(j,k\right)}^{\ell_{2}}$), with weight $t_{E}(e_{\left(i,j\right)}^{\ell_{1}})$ (respectively, $t_{E}(e_{\left(j,k\right)}^{\ell_{2}})$). At node *j*, a transfer is possible between the two lines, which is represented by a link with weight *t*_*T*_(ℓ_1_,ℓ_2_,*j*) corresponding to the actual time of transfer.

Formally, the public transportation system is defined as a weighted, directed, edge-labelled graph *G*=(*V*,*E*,*t*_*E*_,*T*,*t*_*T*_) with vertex set *V* with cardinality *N*, corresponding to the public transportation stops, edge set *E* with weight function *t*_*E*_, and set of transfers *T* with weight function *t*_*T*_. If a line ℓ_*k*_ is defined as an ordered sequence of stops connected consecutively, in the corresponding P-space graph *G*, there will be a direct labelled-edge $e_{ij}^{\ell_{k}}\in E$ connecting each pair of nodes (*i*,*j*) on the given line, such that stop *i* precedes stop *j* in the sequence of line ℓ_*k*_. This way each transportation line appears as a fully connected clique in the P-space representation. We define *M* as the total number of lines in the PT system. Furthermore, a set *T*⊂*M*×*M*×*N* of transfers identifies triplets of two lines and one node, $e_{\ell_{1},\ell_{2},j}^{T}=(\ell_{1},\ell_{2},j)$ assigning a possible transfer between lines ℓ_1_ and ℓ_2_ at station *j*. Each edge in *E* is weighted by the average travel time on the actual line. It is computed through a *time* function $t_{E}:E\to R^{+}$, quantifying for each edge $e_{ij}^{\ell_{k}}$ the time needed to get from *i* to *j* along the line ℓ_*k*_ averaged on a selected time window [*h*1,*h*2] over *N*_*w*_ weeks. The travel time assigned to an edge $e_{ij}^{\ell_{k}}\in E$ is then calculated as the sum of the average waiting time and the average time spent on the vehicle as

2.1 $$t_{E}(e_{ij}^{\ell_{k}})=\frac{1}{2f_{\ell_{k}}}+\Delta t_{ij}^{\ell_{k}},$$

where *f*_ℓ_*k*__ is the average frequency of line ℓ_*k*_ and $\Delta t_{ij}^{\ell_{k}}$ is the average time one needs to spend on line ℓ_*k*_ to go from stop *i* to stop *j*. This formula is designed to consider the case where a user would go blindly to a stop (without looking at the schedule). Another approach would include that certain passengers attempt to reduce their waiting time by timing their arrival at transit stops to an optimal period before vehicle departure. Most studies report that passengers facing short headways or low reliability do not generally pursue these strategies [40–42]. Hence, we choose our approach to favour lines with high frequency and less variability due to unexpected perturbations while accounting for preference of users for low unexpected variability in the total travel time. Finally, the transfer time function $t_{T}:T\to R^{+}$ quantifies for each transfer $e_{\ell_{1},\ell_{2},j}^{T}$ the time needed to change between lines ℓ_1_ and ℓ_2_ at node *j*.

In such description, the temporality of the system is included through the weights. The choice not to model the system as a temporal graph is motivated by the fact that in urban public transportation systems the total travel time is subject to variability and this factor matters considerably for the user when deciding to opt for public transportation service.

### 2.2. Uncovering efficient transportation connections

The previously defined public transportation graph *G*=(*V*,*E*,*t*_*E*_,*T*,*t*_*T*_) is used to calculate the shortest time paths between stops. In the multi-edge representation a path is defined as a sequence of edges^3^ $P_{E}={\left\{e^{\ell_{i_{1}}},e^{\ell_{i_{2}}},\ldots ,e^{\ell_{i_{n}}}\right\}}_{o,d}$ connecting an origin node *o* to a destination node *d* through a sequence of consecutive trips made on *n* lines, ℓ_*i*_1__,ℓ_*i*_2__,…,ℓ_*i*_*n*__. Considering also the sequence of corresponding transfers between lines $P_{T}={\left\{e_{\ell_{i_{1}},\ell_{i_{2}}}^{T},e_{\ell_{i_{2}},\ell_{i_{3}}}^{T}\ldots ,e_{\ell_{i_{n-1}},\ell_{i_{n}}}^{T}\right\}}_{o,d}$ the shortest time paths between origin and destination are taken as the smallest durations measured among the different alternative paths. Each time length is defined as

2.2 $$L_{P}=\sum_{j=1}^{n}t_{E}(e^{\ell_{i_{j}}})+\sum_{j=1}^{n-1}t_{T}(e_{\ell_{i_{j}},\ell_{i_{j+1}}}^{T}),$$

i.e. the sum of the average time needed to wait, travel and transfer between lines.

We adapted the Dijkstra algorithm [33] to provide approximated shortest path lengths between any pair of stops in the user-based multilayer representation, while keeping the interpretable description of the PT system and reduced computation time. The original version of the algorithm computes the minimal distance between any origin *o* and destination *d* nodes by considering the sum of link weights. Instead, the modified version accounts for the fact that not only the link weights have to be taken into consideration, but also the transfer time, i.e. the cost to change between different layers (see the electronic supplementary material, section S3 for more details). Also, to consider the preference of users to change lines a limited number of times, the algorithm allows at most two transfers in a single path, i.e. we limit *n*≤3. Owing to these limitations, the algorithm provides us an approximate solution, which, however, differs from the correct solution in only few cases. We find that more than 95% of the paths with at most three line changes computed with the unlimited (correct) algorithm and the limited (approximate) algorithm have the same temporal length in all cities (see the electronic supplementary material). After computing the shortest paths between all nodes in the graph, we characterize the distribution of shortest travelling times between all nodes whose physical distance falls within a specific range. Using this information, we identify privileged connections, i.e. fastest routes at a given distance.

### 2.3. Implementation of the user-based representation

The methodology presented above relies on information, which is typically included in data given in GTFS format (https://developers.google.com/transit/gtfs/reference) such as trips, routes, travelling times, frequencies and transfer times recorded for each service line and station in the transportation system (for further details, see the electronic supplementary material, section S1). Using such data, we build the P-space multi-edge representations of greater Paris, Strasbourg, Nantes and Toulouse. We decided to use a period of *N*_*w*_=4 weeks in each case, such that the total number of trips per day presents only weak fluctuations. We were interested in trips planned between *h*1=7.00 h and *h*2=10.00 h (though the choices of *N*_*w*_, *h*1, and *h*2 are adjustable parameters). This choice of time window was made to focus on morning commuting patterns, and because during this time interval, the frequency of services is considerably higher than for the rest of the day. Typical line frequencies and trip durations are then defined as their averages over the selected time window over the four weeks. All PT systems considered rely substantially on three transportation modalities: metro (Paris, Toulouse) or tram (Nantes and Strasbourg), bus and rail. However, they differ considerably in terms of size (see the electronic supplementary material, table S2), route length, number of stops per route and route frequencies (see the electronic supplementary material, figure S1).

Finally, building on the multi-edge P-space representation and the estimation of the typical times and frequencies, we compute the typical shortest time paths between pairs of origin and destination in the city. The implementation of this methodology is available online (https://github.com/lalessan/user_basedPT) and requires as input any dataset in GTFS format and parameters summarized in the electronic supplementary material.

## 3. Illustration: fingerprints of public transportation networks

We demonstrate one possible use of our framework through the examples of the PT systems of greater Paris, Strasbourg, Nantes and Toulouse. After selecting privileged connections, we apply non-negative matrix factorization (NMF) to the graph of the privileged connections to identify underlying patterns, which may not be present due to overall design. Finally, we compare our findings with independent measures of commuting patterns, which allow us to give an estimation of the efficiency of the PT systems.

### 3.1. Selection of efficient connections

**Figure 2.**
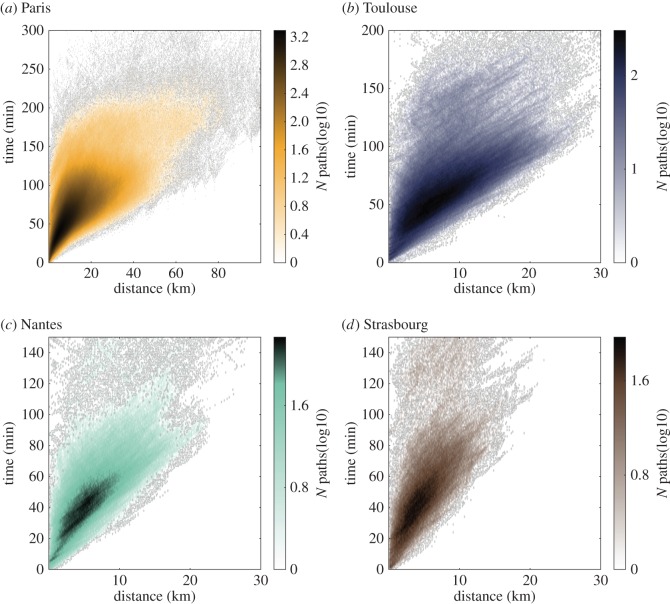
Scatterplot of time versus physical distance associated with the shortest time paths for each origin–destination pair. The points are coloured according to the number of points in the area considered. Scatterplots are shown for the cities of (*a*) Paris, (*b*) Toulouse, (*c*) Nantes and (*d*) Strasbourg. Colours indicate the logarithm of the number of origin–destination pairs in a given range time–distance bin.

We used the method previously presented to compute the shortest time paths for all origin–destination pairs of the transportation systems of bus, train and metro. With the selection of a suitable time-window of size *N*_*w*_=4 weeks, we find that the average standard deviation of a route frequency across the 4 weeks is about 0.05 transits/hour for all the cities considered (focusing only on weekdays). This result confirms that the system behaviour is subject to low variability during the period considered. Based on the shortest path calculations, we built a time–distance map, which assigns the physical distance *d*(*o*,*d*) and the shortest time path length *Δt*(*o*,*d*) to each origin (*o*)–destination (*d*) pair. This time–distance map was drawn as a heat-map in figure 2 for Paris and the other cities investigated, and can be used to identify patterns of privileged connections. We considered distance-bins with equal size 100 m and time bins of size 1 min.

**Figure 3.**
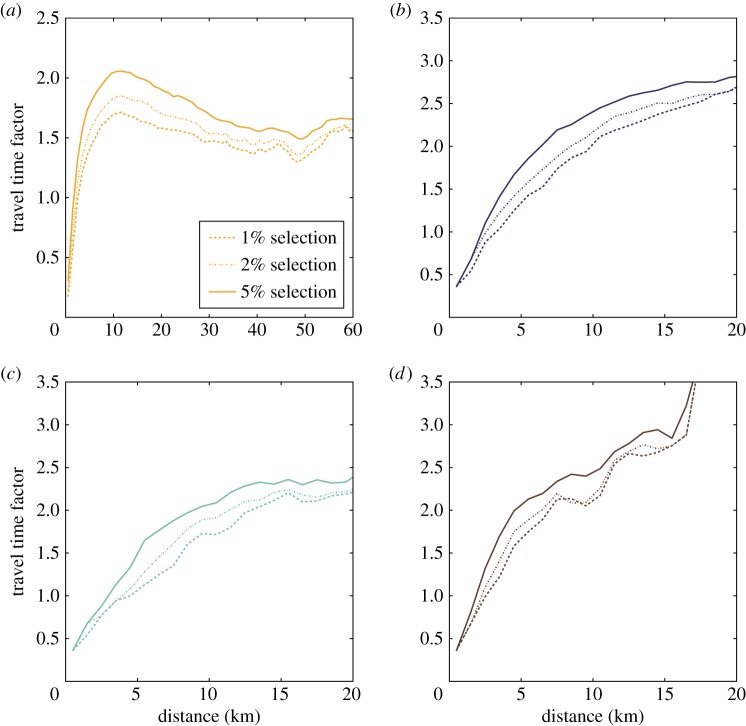
Travel time factors with respect to distance travelled. The factors have been computed using the lowest 1, 2 and 5% of the time distribution for each distance travelled by public transportation for the following cities (including their surrounding areas) (*a*) Paris, (*b*) Toulouse, (*c*) Nantes and (*d*) Strasbourg.

In order to focus on the most efficient (privileged) connections with respect to the public transportation system of the city considered, we selected the trips responsible for the lowest 1% of the time distributions for each distance. To estimate whether these connections are among the best at the urban agglomeration level when compared with travel by car for the same distances, we computed the travel time factor. More precisely, after building the histogram of the shortest time paths for every distance bin, we compared the travel time of selected paths with the travel time needed to cover the same distance by car. Car commuting times were extracted from the French 2008 Enquête Nationale Transports et Déplacements 2007–2008 dataset [43]^4^ describing the global mobility of people living in France. To collect these data, individuals were asked how far (with resolution of 1 km), how long (with resolution of 1 min) and by which means of transportation they travel every day. Based on this dataset, we computed the median of the travel time distributions at each distance using the entire sample to measure the typical time needed to commute a particular distance by car. Similarly, we calculated the medians of the best 1, 2 and 5% of the time distribution at each distance (i.e. shortest times for a given distance) travelled by public transportation. This enables to compute the travel time factor as displayed in figure 3 for different selections of the best times taken by public transportation. By selecting the best connections responsible for the lowest 1% of the time distributions for each distance, in Paris agglomeration, we found that trip durations are at most 1.71 times the time needed by car. This is in close agreement with the travel time factor tolerated by users [34], which was shown to be maximum 1.6 in [34]. For the other agglomerations studied, the travel time factor goes above this value for distances travelled greater than 5 km. We note that while in Paris the travel time factor tends to saturate at large distance, meaning that efficient connections exist also at the inter-city level, this is not the case for the other cities (figure 3*b*–*d*), where PT seems to provide an efficient alternative to car mainly for short trips.

Let us notice again that in the histograms and travel time factor calculations, we do not use the best absolute time to travel a given distance but we consider a waiting time assuming that a user arrives blindly at a stop, in order to take into account the preferences of users for paths with small variability in time. In addition, the time travelled by car for each distance is taken from data considering car trips in the whole country. These two points may lead to an overestimation of time travel factors, so the travel time factor cannot be used directly as a criterion to select the best connections but only gives a common metric to look at the different public transportation systems.

For all the cities considered, privileged connections include shortest paths with no line changes at very short distances, and an increasing number of line changes as a function of the distance travelled (see the electronic supplementary material, figure S6). In the Paris area, only two transportation modalities are used in 80% of the paths up to 60 km distance and the most-represented modalities include metro, bus and rail, with metro dominating at short distances. In smaller cities, almost all paths at distances up to 20 km involve less than two changes. The most-represented modalities are bus and tram for Strasbourg and Nantes, bus and metro for Toulouse; instead, rail is present only when the path distance is larger than 15/20 km (see the electronic supplementary material, figures S7 and S8).

**Figure 4.**
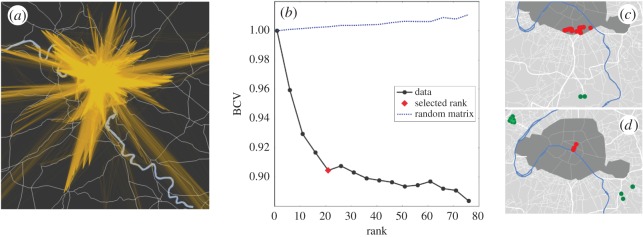
Pattern detection using the multi-edge P-space representation. (*a*) Geographical representation of graph *G*_SP_, where links correspond to the 1% best shortest paths of the whole public transportation network. (*b*) The normalized BiCross validation error computed for the adjacency matrix *X*_SP_(10 km,11 km) (solid black line) of the same graph, for the associated random matrix *X*_SPrandom_(10 km,11 km) (dotted line). The selected number of structures *k*_s_ is assigned by a red rhombus. (*c*,*d*) Two of the structures revealed in the PT system of Paris. Green dots are ingoing, while red dots are outgoing affiliated.

In figure 4*a*, we show the profile of the Paris urban agglomeration, where links correspond to the selected privileged connections. The city profile differs clearly from the profile obtained for single-modality single-layer representations, since it accounts for the interconnectedness of several transportation modes (see the electronic supplementary material, section S7). Note that, since we do not have access to the transfer times between lines in cities other than in Paris, the cost of transferring between layers was estimated for each city based on the data for Paris (see the electronic supplementary material, table S3). This way, transfer times depend on the corresponding transportation modes, which a naive representation with all modes on a single layer would not be able to consider.

### 3.2. Pattern extraction

The question remains whether the identified set of privileged connections reveals any higher-order meaningful patterns in the design of transportation systems. We expect that some stops, such as stations located in residential neighbourhoods, may have similar connectivity patterns to the rest of the network as to the city centre or to working areas. In order to identify such patterns, we first built an undirected, unweighted graph *G*_SP_=(*V*_SP_,*E*_SP_), where *V*_SP_⊂*V* and *E*_SP_ are a set of edges linking origin–destination locations connected by privileged connections (for an example for Paris, see figure 4*a*). To compare commuters travelling at particular distances, we analysed subgraphs *G*_SP_(*d*_1_,*d*_2_) (represented by an adjacency matrix *X*_SP_(*d*_1_,*d*_2_)) of *G*_SP_, where edges join stops at particular distances *d* (*d*_1_<*d*≤*d*_2_). For Paris, we considered distances with resolution *d*_2_−*d*_1_=1 km, while for smaller cities we took the resolution *d*_2_−*d*_1_=5 km as the transportation networks were typically sparser there (see the electronic supplementary material, figure S1).

We expected to find both cohesive and bipartite patterns in these subgraphs. The cohesive structures would correspond to sets of stations well connected among themselves, while bipartite ones would single out two groups of stops with several connections between them. The connections may not be direct but should have durations comparable to the average time taken by car for the same distance.

To detect such patterns, we considered the likelihood of having a connection between any two stations, which can be expressed in terms of possible connections of these stations to the same structures. Formally, it means we can express each term of the adjacency matrix representing *G*_SP_ as

3.1 $$X_{\mathrm{SP}}(i,j)=\sum_{k}W_{ik}H_{kj},$$

where *W*_*ik*_ quantifies the ingoing membership of node *i* to structure *k* and *H*_*kj*_ quantifies the outgoing affiliation of the node *j* to the structure. In order to find matrices **W** and **H**, we performed matrix factorization, thus minimizing numerically the distance

3.2 $$∥\text{X}-\text{WH}{∥}_{F}^{2},$$

where ∥**X**∥_*F*_ is the Frobenius norm of matrix **X** (for further details see the electronic supplementary material, section S2). Note that matrix factorization was used earlier successfully to detect communities and higher-order structures in graphs [44–50].

The number of structures to be detected was determined by the Bi-Cross validation (BiCv) approach proposed in [51] based on cross-validation, a common machine learning model validation technique. This consists of measuring an error, called BCV here, between an estimation of left-out entries using a low-rank approximation of the retained data and the actual left-out entries. This error is decreasing with respect to the number of structures extracted towards a minimum that indicates how many structures are representative of the subgraphs (more details in the electronic supplementary material, section S4), while, on the contrary, such behaviour is not visible when the network is close to random. To identify whether there are structures in subgraphs, we compared the BCV error with the one obtained for the corresponding null models (figure 4*b*). Such null models were defined for each adjacency matrix *X*_SP_(*d*1,*d*2) as their corresponding random matrices *X*_SPrandom_(*d*1,*d*2) with the same size and density. An example of the behaviour of such a quantity for the Paris public transportation network is displayed in figure 4*b* (for other cities, see the electronic supplementary material, figure S1). This quantity was computed for each subgraph and guided us on how many structures characterize each system at each range of distance. For some distance ranges and cities, the evolution of BCV is close to the random case assigning no strong attempt to link preferentially some areas at the considered range of distance (see the electronic supplementary material, figure S2). However, we find bipartite structures in several cases, like the two examples in figure 4*c*,*d* detected in the Paris network. The bipartite structures can be assimilated to strategical areas that are particularly well connected by PT. For example, the structure shown in figure 4*c* connects stops located around Paris Orly airport to stops located at the border of the Paris central area. In figure 4*d*, the structure reveals the existence of privileged connections between the Nanterre and Creteil areas on one side (both with high employment density; http://insee.fr/fr/themes/document.asp?reg_id=20&ref_id=20718&page=alapage/alap417/alap417_carte.htm#carte1) and Paris centre on the other side. As these structures are latent patterns extracted from the networks of privileged connections, we consider them as the privileged origin–destination patterns representative of the transportation systems.

### 3.3. Network efficiency: pattern analysis from the commuter point of view

To estimate how well the different public transportation networks are devoted to answer the needs of commuters, we compared the identified privileged origin–destination patterns to the flows of commuters. We used the data of the 2010 French census [52] including origin–destination commuter flows per means of transportation at the level of the municipality for the areas of greater Strasbourg, Toulouse and Nantes, and at the level of the municipal arrondissement (neighbourhood) for the Paris agglomeration. Using this dataset, we compared the detected privileged origin–destination patterns to the commuting patterns by car and PT. We only considered inter-municipality trips for the comparison as the resolution provided for the commuter dataset was given at the municipality level (for the number of intra-city trips, see the electronic supplementary material, table S4).

To draw a comparison, we first built the PT structural pattern network *G*_*C*_=(*V*_*C*_,*E*_*C*_) of each urban agglomeration as an unweighted, undirected graph. Here, the set of nodes *V*_*C*_ is defined as municipalities and a link (*a*,*b*)∈*E*_*C*_ between municipalities *a* and *b* exists if at least one stop located in *a* and one stop in located *b* appear in each side of a detected bipartite structure. In other words, the structural pattern networks are composed of links between municipalities presumably well connected by public transportation. At the same time, exploiting census data, we built a commuter flow network for each city and its surrounding area, as a weighted, directed graph $G_{\mathrm{com}}^{TM}=(V_{\mathrm{com}}^{M},E_{\mathrm{com}}^{TM},W_{\mathrm{com}}^{M})$. Here, *V*^*TM*^_com_ is the set of municipalities, and a link $(a,b)\in E_{\mathrm{com}}^{M}$ with weight *w*_*ab*_ represents the flow of individuals commuting from *a* to *b* by means *TM* (either PT or car). We compared the structural pattern graph with the commuter flow graphs both of the car and the PT of each urban agglomeration by computing a weighted Jaccard index *s* between the sets of links associated with each graph. This weighted index is defined as the sum of the flow graph weights of the links in common between the two graphs—structural and flow by the selected transportation means—divided by the total flow for the transportation means considered. More formally

3.3 $$s_{TM}=\frac{\sum_{(a,b)\in E_{C}\;\cap \;E_{\mathrm{com}}^{TM}}w_{ab}}{\sum_{(a,b)\in E_{C}\;\cup \;E_{\mathrm{com}}^{TM}}w_{ab}}$$

both for *TM*=*car* and *TM*=PT. It represents the fraction of commuters using, respectively, car and PT, who have access to privileged PT connections (i.e. for which there exists a link corresponding to their commuting in the PT structural pattern).

**Figure 5.**
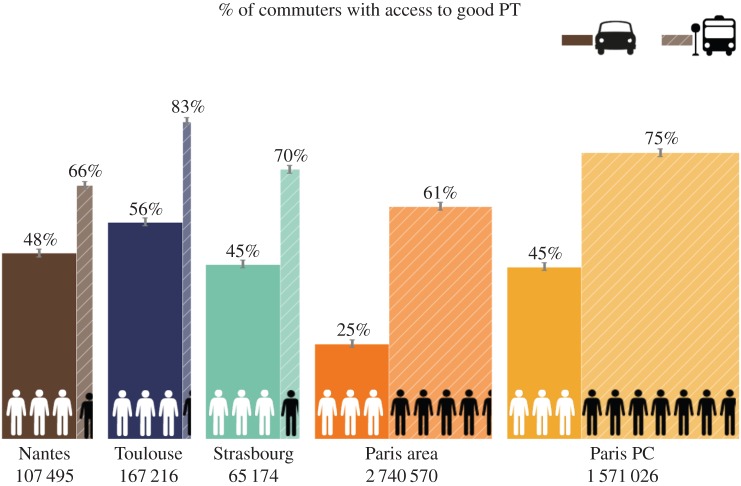
Similarity between commuter flows and PT privileged connections in French municipal areas. For each of the urban agglomerations considered (Toulouse, Nantes, Strasbourg, Paris area and Paris Petite Couronne), the bar chart’s height indicates the weighted Jaccard index *s*_*TM*_ between the commuter flow network *G*^*TM*^_com_ and the PT structural pattern network *G*_*C*_ (for further explanation see text). The total number of commuters within each city using either car or PT is indicated below each bar.

The bar charts in figure 5 show the comparison between commuting flows and privileged connections for several urban agglomerations. Full bars refer to commuters choosing the car, while dashed bars refer to the choice of PT. The width of full bars are set to be equal for all the cities considered, and the width of the corresponding dashed bar is set proportionally. Hence, for each city the number of black (respectively, white) stick men over the total number of stick men corresponds to the fraction of individuals choosing to commute by PT (respectively, car). The total number of commuters (using either car or PT) in each city is indicated below each bar. For example, in the Paris central agglomeration (Paris Petite Couronne (PC), on the right of the figure), there are about 1.6 million commuters using either car or PT. Among them, for every three commuters choosing the car, about seven choose PT.

The height of the full bars (respectively, dashed filled) assigns the weighted Jaccard index *s*_car_ (respectively, *s*_PT_), indicating the fraction of individuals choosing the car (respectively, PT) even with access to privileged PT connections. For example, in the Paris central agglomeration, among all commuters choosing the car only 45% would have access to privileged connections, while among those who are choosing PT, 75% can rely on efficient transportation. Error bars on the top of the bars are obtained by repeating the methodology 100 times, with different random matrices initializing NMF. The small size of the error bars shows the robustness of the pattern detection.

A significant difference between the commuting practice in Paris agglomeration and other urban areas is evident. For Paris urban agglomeration, the flow of inter-municipality commuters choosing PT is larger than that of people commuting by car, in contrast to the other cities investigated. This may be partly explained by a travel time factor, which increases above the tolerated value for Toulouse, Nantes and Strasbourg (figure 3). Besides, figure 5 indicates that the fraction of commuters having access to privileged connections and actually using the PT systems is larger than the fraction of them using the car for all urban agglomerations studied. This supports our definition of privileged connections based on commuting time with little variability and a limited transfer number. This corroborates the strong role of the latter factors in the decision-making to use PT or car. Furthermore, we observe that in the greater Paris area only 25% of car commuters have access to privileged transportation connections. Instead, in other cities, although more than 48% of car drivers have access to rapid connections, they still commute by car. In particular, in Toulouse a large percentage of commuters have access to good services according to the criteria introduced here, as there is large overlap between privileged connections and both PT (83%) and car (56%) commuting flows. However, there is still a non-negligible amount of people commuting by car. Based on this analysis, we can distinguish between two main trends in commuting: (i) there are cities where a large part of the population tend to do inter-municipality trips by car disregarding the quality of PT services, examples are Nantes, Toulouse and Strasbourg. (ii) On the other hand, in Paris and its agglomeration, according to the metrics introduced, there is a good agreement between the needed and provided services of public transportations. This result is supported by a pairwise comparison between the car and the PT commuting flows for every pair of municipalities (see the electronic supplementary material, section S5).

## 4. Conclusion

Efficient analysis of public transportation networks is possible via abstract representations, which in turn help us to reveal hidden characteristics of such systems. As our main scientific contribution we provided a solution for this challenge by introducing a novel description, which combines multi-edge and P-space representations of multilayer transportation networks. We characterize these systems from the user’s point of view through a description, which is detached from constraints imposed by their spatial embeddedness, but which incorporates their temporal variance. To further develop our framework, we adjusted earlier-defined methods and used them to identify effective routes and hidden transportation patterns, which were not evidently built due to overall design. We found cohesive and bipartite patterns of privileged connections induced by different ways of access of far-apart urban areas in French municipals such as greater Paris, Toulouse, Nantes or Strasbourg. We further analysed the overall efficiency of the corresponding transportation systems when compared with the commuting flow. We found that while the transportation system of Paris is somewhat meeting overall demands and its use is preferred over the car alternative, in smaller cities, the transportation systems may not meet user expectations, leaving room for improvement, and even people with access to fast transportation options prefer to use car instead.

We made some assumptions during our study, which set some limitations on the generalization of our results. First of all, we considered only a 3 h time window to build our user-based representation. Extending this time window or considering different periods would potentially highlight further transportation patterns, assigning a direction to explore in the future. Furthermore, we operated with average frequencies of services neglecting the effect of any perturbation in the transportation system. This was a valid approach in our case as no major variance was observed during the analysed period. Nevertheless, to get around this limitation one can easily adjust our definition such that it considers dynamically unexpected perturbations on each line. Finally, we assumed that passengers go blindly to a stop without considering that certain passengers attempt to reduce their waiting time by timing their arrival at transit stops to an optimal period before vehicle departure. On the other hand, this can be easily considered in our representation by introducing arrival times of users, e.g. depending on the frequency of the first line they take. In addition, note that aspects such as adaptive travelling behaviour or the prediction of individual mobility patterns are out of the scope of the present methodology but they indicate possible future directions of research.

Several extensions of our methodology are possible. Parameters like the periods in focus, length of observations, number of transfers, etc., can be tailored for other systems, while a further refinement is possible by considering needs of various types of users. Our way of characterization of privileged connections may be used to profile and compare different transportation systems to disclose generalities in their design. The use of this methodology in the future could help to enhance resilience of local transportation systems to provide better design policies for future developments.

## Supplementary Material

Supplementary Information

## Footnotes

1 This document provides a study on the factors influencing the choice of the transportation means. The study is based on a literature review and statistical analysis of surveys.

2 This document presents results of a survey about transport and mobility of households in France. The authors consider the distance travelled, if it is a long-distance trip or short trip made on a daily basis. Moreover, they look at the differences of behaviours in transportation use among different regions and individuals with different socio-demographic characteristics.

3 In the current paragraph, to simplify notations, we do not index edges by node names.

4 This reference links to a file about the home–work flow of individuals by transportation means.

## Data accessibility

All data used in this paper are openly shared by the local transportation companies or central statistical institutes. The datasets used can be found within an extensive list of similar datasets collected in [16,17], while the census data used can be found in [43,52]. The source of the implementation of our methodology is openly shared under the link https://github.com/lalessan/user_basedPT.
