# Supplementary material for: User-based representation of time-resolved multimodal public transportation networks
Source: R Soc Open Sci. 2016 Jul 13;3(7):160156. doi: 10.1098/rsos.160156 (PMC4968465; doi:10.1098/rsos.160156)
Supplement: Supplementary Information [file rsos160156supp1.pdf]

Supplementary Information for

User-based representation of time-resolved multimodal public transportation networks

Laura Alessandretti, Márton Karsai and Laetitia Gauvin*

*Corresponding author email: marton.karsai@ens-lyon.fr

Contents

S1 Data description **2**

S1.1 Coarse graining network stops . . . 2

S1.1.1 Paris . . . 3

S1.1.2 Nantes . . . 3

S1.1.3 Toulouse . . . 3

S1.1.4 Strasbourg . . . 4

S1.2 Choice of a representative day . . . 4

S1.3 The INSEE datasets . . . 5

S1.4 Matching the INSEE datasets and the PT datasets . . . 7

S2 Structure detection with Non-Negative Matrix Factorisation **7**

S3 The modified Dijkstra algorithm **8**

S4 Pattern detection for Strasbourg, Nantes, and Toulouse **10**

S5 Comparison of the patterns detected and the commuter flows **10**

S6 Characteristics of privileged connections **12**

S7 Comparison with the single-layer representations **12**

S1 Data description

With the aim of catching a comprehensive picture of the public transportation (PT) networks in French municipal areas we made use of datasets provided by local public transportation companies. The characteristics of the datasets used for the different cities are listed in Table S1. Estimated timetable schedules for the public transport service are made publicly available online and frequently updated by the companies.

City	Area	Period	Companies
Paris	47.96N-49.45N 1.15W-3.51W	Sep-Oct 2013	RATP (Bus, Metro, Tram, RER)<br>SNCF (RER,Train)
Toulouse	43.43N- 43.74N 1.17W- 1.69W	Sep-Oct 2014	Tisséo (Bus, Tram, Metro)<br>SNCF (Train)
Nantes	47.12N- 47.32N 1.75W-1.34W	Jan 2015	Semitain (Bus, Tram, Ferry)<br>SNCF (Train)
Strasbourg	48.46N- 48.68N 7.60W-7.83W	Jan 2015	CTS (Bus, Tram)<br>SNCF (Train)

Table S1. Table listing the main characteristics of the data used for each of the cities

All datasets are provided in General Transit Feed Specification (GTFS) format [1]. GTFS is a common format for PT schedules and associated geographic information. It is composed of a series of text files: stops, routes, trips, and other schedule data. In particular, the following objects and associated attributes are of relevance to the purpose of this study:

- **stop**: the physical location where a vehicle stops to pick up or drop off passengers. It is associated to a unique *stop_id* and it has attributes *stop_name*, *stop_lat*, *stop_lon*, respectively the name and the geographic coordinates. (Example: 4025460, "PONT NEUF - QUAI DU LOUVRE", 48.858588, 2.340932)
- **route**: a public transportation line (in the following we refer to "line" or "route" as interchangeable terms) identified by a unique *route_ id*. It has attributes *route_ type*, identifying the type of vehicle, and *route_ name*. (Example : 831555, metro, "14"). Note that the two directions of a same service are identified by two different routes, and that services with multiple termini are identified by several different routes.
- **trip**: a journey of a vehicle, identified by a unique *trip_id*. It refers to the unique route of the actual line, and also to a set of dates indicating in which days of the year that trip is running. It is also associated to an ordered sequence of stops of the vehicle, and with the list of arrival and departure time at each stop.

  Example:

trip_id	stop_id	arrival_time	departure_time
1013644000942075	4025388	16:10:00	16:10:00
	4025390	16:11:00	16:11:00
	4025392	16:12:00	16:12:00
	4025393	16:13:00	16:13:00
	...	...	...

S1.1 Coarse graining network stops

To model the transportation network, it was necessary to coarse grain the data by grouping nearby stops together. Table S2 summarises the information contained in each of the datasets before and after coarse-graining.

Area	Stops	Routes	Train stops	Train routes	Tot stops after merging
Paris	11850	1058	494	169	5690
Toulouse	1913	106	59	31	1920
Nantes	3412	61	27	18	1038
Strasbourg	1330	53	31	17	601

Table S2. Table illustrating the main characteristics of the PT systems datasets. For each urban agglomeration (Area), we indicate the number of Bus, Metro, Tram and RER stops and routes before coarse graining (Stops, Routes), the number of train stops (Train stops) and routes (Train routes), the total number of stops after coarse-graining and merging the two datasets (Tot stops after merging).

S1.1.1 Paris

The transportation system described in the RATP dataset contains 11850 stops. Some of these stops closely located to each other can be functionally replaced by a single station via a careful merging method. In order to merge stops, we used the information provided in the GTFS dataset. Data provides the list of stop pairs that are located at a short distance from each other, allowing people to transfer walking, from one route to a different one in a given amount of time (that is also given in the dataset). It is for example the case of main railway stations or big squares, where many stops are concentrated in a relatively small area. We merged corresponding stops according to the information provided by the RATP company on possible transfers, as well as bus stops located in front of each other at the two opposite sides of the same road. After coarse graining, the total number of stops for the RATP dataset was reduced to 4596.

In the SNCF dataset, there is a total number of 494 suburban railway stations. It is necessary to identify stops/train stations present both in the SNCF and RATP datasets (i.e "Gare du Nord" is both a RER station and a metro stop). To do so we built a grid with a resolution of 0.25 Km and we identified for each of the train stations the cell it belongs to. A train station was then identified by the closest RATP stops present in the actual cell or in neighbouring cells otherwise. In the city centre, all the train stations were identified with RATP stops, while in the suburbs it was not always the case.

S1.1.2 Nantes

The Semitain dataset contains 3412 stops. It indicates for each stop whether it is part of a larger station complex (stops that are located on the opposite side of a same road are considered part of a unique station). Using such information, it was straightforward to merge close-by stops. Since transfer time was not provided, we estimated the time to change line based on the data provided by RATP (average transfer time, see table S3). After coarse graining, the network includes 1036 stops. The SNCF dataset was used to include the train stations which are located in the area served by the Semitain company. Using the same method we used for Paris, we found their corresponding stops in the Semitain dataset.

S1.1.3 Toulouse

The Tisséo dataset contains 5694 stops. As in the case of Nantes, the Tisséo dataset provides information on parent stations. We merged stops accordingly received 1913 stops in total. Since transfer time was not provided, we estimated the time to change line based on the data provided by RATP (average transfer time, see table S3). From the SNCF dataset, we selected 59 stops that located in the same area served by the Tisséo company.

S1.1.4 Strasbourg

The CTS dataset contains 1330 stops. Even if it does not provide information on parent station, we could merge stops based on their *stop_id*. Since transfer time was not provided, we estimated the time to change line based on the data provided by RATP (average transfer time, see table S3). In fact, in this dataset all stops that are part of a larger station complex have the same name and in addition a unique number (Example: stops {DANTE_01, DANTE_02, DANTE_03} are part of a same large station complex). After coarse graining this way 595 stops were identified in the CTS dataset. From the SNCF dataset, we selected 31 stops that are located in the same area served by the CTS company.

Mode 1	Mode 2	Average transfer time (sec)
bus	bus	70
subway	rail	326
tram	rail	222
rail	rail	60
subway	bus	230
tram	bus	92
subway	subway	172
rail	bus	232
tram	subway	212
tram	tram	66

Table S3. Average transfer time (in seconds) between different transportation modalities

S1.2 Choice of a representative day

The datasets provide the schedule over several months in normal situations (which means no perturbation due to traffic jams or to system breakdowns) with a 1-minute resolution. We do not consider exact travel time at a given departure time but an estimation of the time taken in a "typical" day. The description of a typical day is given below.

In order to draw typical commuting times we first selected a window of $N_w = 4$ consecutive weeks. A week $w_i = \{d_1, d_2, d_3, d_4, d_5\}$ is defined as a set of five consecutive days, from Monday to Friday. The separation week-end/week days is necessary as the system behaviour is different in these two cases. For every span of consecutive weeks $W = \{w_1, w_2, w_3, w_4\}$, we calculated the average daily number of trips $\langle Nt_W \rangle = \sum_{d \in W} Nt_d / D$. Here D is the number of days ($D = 5 \times 4 = 20$), Nt_d is the number of trips during day $d \in W$. Then, by looking at fluctuations from the average $\sigma_W^2 = \sum_d (Nt_d - \langle Nt_W \rangle)^2 / D$, we selected the four weeks span W for which σ_W^2 is the smallest. For each city the selected period is indicated in Table S1.

The reason to select a span of time where the number of trips is not fluctuating is motivated by the need to work with meaningful averaged quantities. We are aware that the results of the illustration may not generalise well, as they are relative to a specific selected period of time. Future work could include a comparison to the system behaviour during weekends, and at different times of the year.

For the purpose of this work, as we aimed at comparing our results with the flux of commuters, we limited the analysis to the 7-10am time interval. Indeed, as a first step, we selected all trips occurring between $h1 = 7am$ and $h2 = 10am$ within the selected period. Further work could include the study of the system evolution at different times of the day.

As a second step, we calculated for each route ℓ_k and each day $d \in W$ the total number $Nt_{\ell_k, d}$ of trips tr occurring on day d between 7 and 10 am and computed its average over the four selected

weeks $\langle Nt_{\ell_k}\rangle = \sum_{d\in W} Nt_{\ell_k,d}/D$. In this way, we received the average frequency $f_{\ell_k} = \langle Nt_{\ell_k}\rangle/3h$ (3h is the length of the time interval) in the selected period for each metro, bus or train line. Also, we computed in equivalent way, the average duration of a trip between any two stops i and j along line ℓ_k: $\langle \Delta t_{ij}^{\ell_k}\rangle = \sum_{tr}(\Delta t_{ij}^{\ell_k})/\sum_{d\in W} Nt_{\ell_k,d}$ considering all selected trips tr.

In figure S1, we show the characteristics of the PT datasets for the 4 cities considered. We observe that stops are highly heterogeneous with respect to the number of routes in all the cities considered, with few highly connected stops and a considerable number of stops served by only one or two routes. The number of stops per route vary greatly for all transportation modalities in Paris; for other cities only buses routes have more than 40 stops, and rail routes are relatively short (up to 10/15 stops). Also service frequencies have large varitations depending on the transportation modality, with metro lines running considerably more frequently than other services (up to $\sim$ 45 times per hour), and rail services running at most $\sim$ 10 times per hour.

S1.3 The INSEE datasets

In order to analyse commuting patterns, we gathered two datasets of the French Institute of Statistics (INSEE): the *Enquête Nationale Transports et Déplacements 2007-2008* [2] used for computing the commuting travelling times, and the 2010 French census (*Recensement de la population 2010*) [3] to extract origin-destination commuting patterns.

We used the file "Q_ind_lieu_teg.csv" of the **first dataset** providing for each individual several informations about their daily journey to work/school. We estimated the average time needed to commute a specific distance by car by scanning over the following variables *V1_BTRAVDIST*, i.e. the distance covered daily (resolution 1Km), *V1_BTRAVTEMPSA* i.e. the time needed to cover such distance (5 minutes resolution), and *V1_BTRAVMOYEN1S*, i.e. the transportation mean used. The time computed for a given distance is the time average over the trips with the same distance and travelled by car.

The flow of commuters for each origin-destination trip was estimated using the file "FD_MOBPRO_2010.txt" of the **second dataset**, in which each line provides several variables related to an individual interviewed. In particular, the following variables were needed: *COMMUNE* and *ARM*, respectively indicating the INSEE code associated to the municipality and the arrondissement (available only for central Paris) where the individual interviewed lives, *DCLT* the INSEE code indicating the municipality and the neighbourhood (only for Paris) of work, and *TRANS* referring to the transportation mean used to commute (either by foot, two-wheeler, car/camion/van, PT). We also considered the variable *IPOND* to take into account that, because not every single citizen is interviewed for the census, each individual has a statistical weight to infer a representative behaviour. Tables S4 and S5 provide an overview on the data for each of the urban agglomerations considered for this study.

Area	Mun	O-D pairs	Tot comm	Car comm	PT comm
Paris	460	61897	4321011	1542640	2017768
Toulouse	89	2319	363679	249642	57269
Strasbourg	57	618	170337	92275	36576
Nantes	26	524	225026	143441	45455

Table S4. For each one of the urban areas considered (Area), the table provides with the number of municipalities considered (Mun), the number of origin-destination pairs travelled by commuters (O-D pairs), the total number of commuters (Tot comm), the number of commuters travelling by car (Car comm), the number of commuters travelling by PT (PT comm).

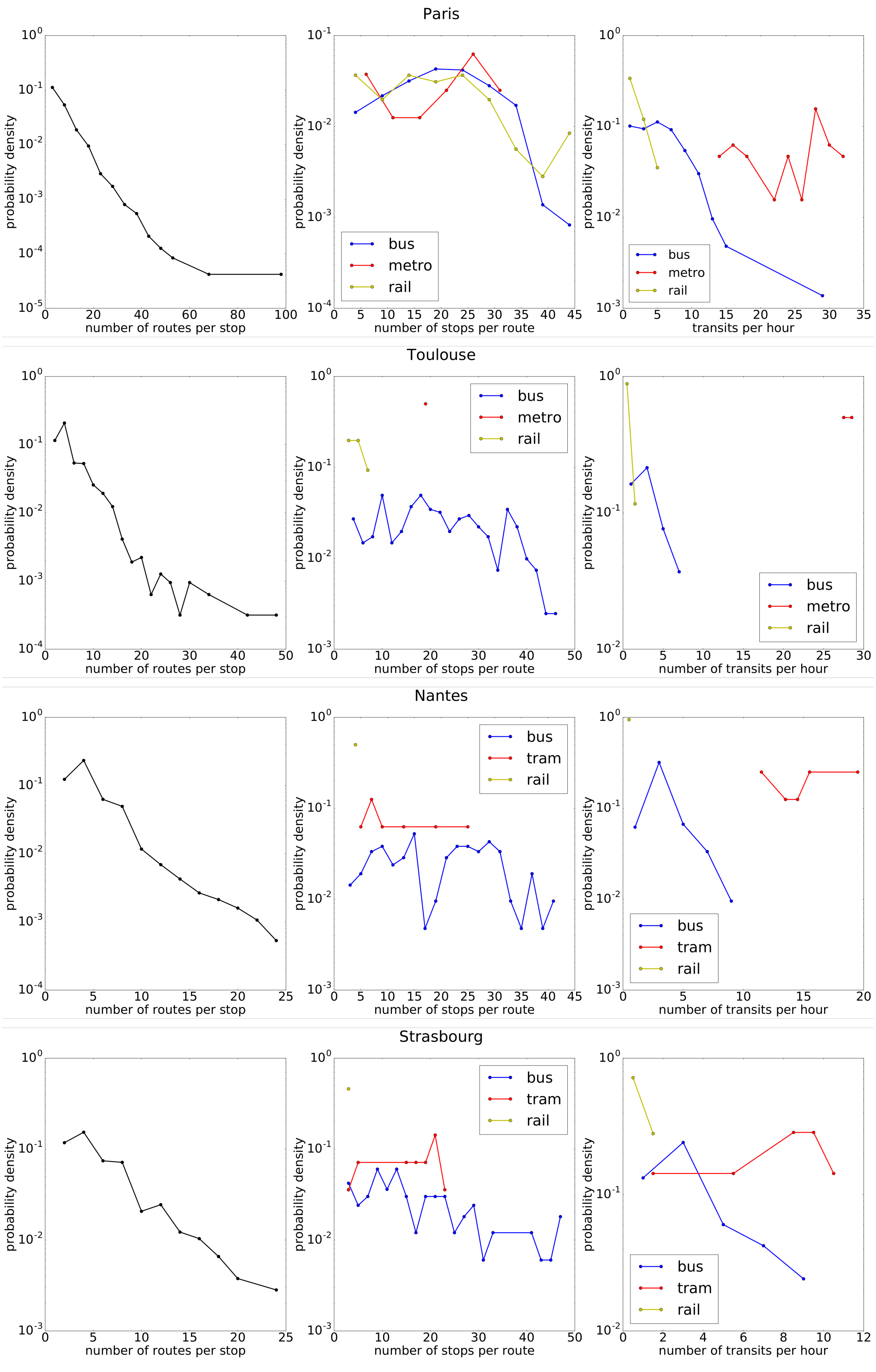

Figure S1. Characteristics of the PT datasets for the 4 cities considered. For Paris, Toulouse, Nantes, and Strasbourg (top to bottom), we show the probability density distribution of the number of routes per stop after coarse-graining (left), the probability density distribution of the number of stops per line, for different transport modalities (center) and the probability density distribution of the number of transits per hour on each route, considering the period between 8 and 10 am (right).

Area	IC comm	IC car comm	IC PT comm
Paris	1369535	390105	429735
Toulouse	187797	99142	40553
Strasbourg	99041	40579	23098
Nantes	111434	55444	25957

Table S5. For each one of the urban areas considered (Area), the table provides with the total number of intra-city commuters (IC comm), the number of intra-city commuters using the car (IC car comm), the number of intra-city commuters using PT (IC PT comm).

S1.4 Matching the INSEE datasets and the PT datasets

In order to establish a comparison between the commuting patterns and the efficient connections of the transportation systems, we matched the INSEE dataset with the PT data by associating to each of the stops in the PT data its corresponding municipality (or neighbourhood in the case of Paris). We made use of the Google Maps API [4] to assign to the latitude-longitude coordinates of each PT stop its corresponding address. Then, we matched the municipality to its corresponding INSEE code via the file *Base communale des aires urbaines 2010* provided by INSEE. [5]

S2 Structure detection with Non-Negative Matrix Factorisation

In this section, we explain non-negative factorisation was achieved in order to extract structures from the transportation system dataset.

Algorithm

Aiming at minimising the Euclidean distance loss function between the original matrix and the factorized one, we implemented the standard multiplicative rule developed by Lee and Seung in [6]:

$$\mathbf{H}_{ci} \leftarrow \mathbf{H}_{ci}\frac{(\mathbf{W}^T\mathbf{V})_{ci}}{(\mathbf{W}^T\mathbf{W}\mathbf{H})_{ci}} \qquad \mathbf{W}_{ic} \leftarrow \mathbf{W}_{ic}\frac{(\mathbf{V}\mathbf{H}^T)_{ic}}{(\mathbf{W}\mathbf{H}\mathbf{H}^\mathbf{T})_{ci}}$$

Initialisation

The NMF algorithm may not converge to the same solution at each run, depending on the initial conditions. To address this problem we initialise the matrices $\mathbf{W}$ and $\mathbf{H}$ randomly and run the algorithm 500 times. At each iteration we compute the divergence $\|\mathbf{V} - \mathbf{W}\mathbf{H}\|_F^2$ and we select the iteration for which the error was minimal.

In the present case, the algorithm turns out to be stable and the results are robust for large networks, future development of this work could however include the study of a consensus clustering procedure. Consensus clustering is the problem of reconciling clustering information about the same data set coming from different runs of the same algorithm. For NMF, some efforts have been done in this directions [7], however, as the result of the clustering is described through two different matrices and the partitioning is soft, the problem is not trivial to solve.

Soft/Hard partitioning

The results of NMF provide a soft clustering of the stops to the structures. Such information is included in matrices $\mathbf{W}$ and $\mathbf{H}$. For a given node i and a given structure k, W_{ik} is the out-going affiliation of node i to structure k, while H_{ki} is the in-going affiliation. As the original matrix can be very sparse, and the NMF algorithm can hardly produce zero-values, many of the values in $\mathbf{W}$ and $\mathbf{H}$ are positive but very close to zero. In order to overcome this problem and to make sure we are capturing only the most relevant information, we applied a method to binaries the matrices $\mathbf{W}$ and $\mathbf{H}$ as follows: For each structure c, vectors $\mathbf{H_c}$ and $\mathbf{W}_c^T$ contain respectively the in-going and out-going affiliation of each node $i \in V$ to the structure c. With the goal of selecting only nodes that are strongly affiliated to c, we applied k-means clustering on these two vectors. k-means clustering partitions the $|V|$ affiliation values into k clusters. By choosing $k = 2$ for each of the structures c we distinguished a subset of not-affiliated nodes, whose affiliation value was very small, and a subset of affiliated nodes, whose affiliation value was significantly different from zero. Using this partitioning we defined a binary matrix $\mathbf{H'}$ such that $\mathbf{H'_{ci}} = 1$ if node i is in-going affiliated to community c and $\mathbf{H'_{ci}} = 0$ if it not. In the same way, we define $\mathbf{W'}$, for the out-going affiliation.

S3 The modified Dijkstra algorithm

We devised a modified version of the Dijkstra algorithm allowing to compute approximated shortest paths in a weighted, labeled-edge graph. Below we present the pseudo code of the modified algorithm.

The algorithm requires:

- A graph $G = (V, E, t_E, T, t_T)$ with vertex set V with cardinality N, edge set E with weight function t_E, and set of transfers T with weight function t_T
- A cut-off L_{max}(the maximal number of line changes allowed)

The algorithm returns:

- An array *dist* of length $N-1$, where $dist[u]$ is the approximated shortest path length between nodes s and u
- an array Π_{node} of length $N-1$, where $p = \Pi_{node}[u]$ is the *parent node* of node u, that precedes it in the shortest path between the source s and u itself
- the array of *parent edges* Π_{node}, of length $N-1$, where $\Pi_{edge}[u]$ is the edge connecting u and its parent node p in the approximated shortest path connecting u and the source s

In the pseudo-code, the following notations are introduced: $lenPath$ assigns to each vertex v the number of edges to reach source s, Q is a min-priority queue initialised with all nodes in V_G, where priority is given to nodes that are at shortest distance from the source s, $EXTRACT-MINQ$ is the operation of selecting and removing the node with highest priority from Q, $e_{uv}^{\ell_k}$ is an edge in E connecting nodes u and v via line ℓ_k, and u is a neighbour of v if at least one of such connections exists and e_m is the edge connecting two nodes in the fastest way, also taking into account possible line transfers when coming from an other node, t_m is the associated time.

Algorithm 1 Dijkstra on P-Space Multiplex network

```
for each vertex v ∈ V_G
    dist[v] = ∞
    Π_node[v] = NIL
    Π_edge[v] = NIL
    lenPath[v] = 0
dist[s] = 0

Q = V_G
while Q ≠ ∅
    u = EXTRACT-MIN Q
    for each v in neighbors u:
        if Π_edge[v] == NIL:
            t_m, e_m = min, argmin(t_E(e_uv^{ℓ_k}))
        else
            t_m, e_m = min, argmin(t_E(e_uv^{ℓ_k}) + t_T(Π_edge(u), e_uv^{ℓ_k}))


        if dist[v] > dist[u] + t_m AND lenPath[u] + 1 <= L_max
            dist[v] = dist[u] + t_m
            Π_node[v] = u
            Π_edge[v] = e_m
            lenPath[v] = lenPath[u] + 1

            return dist, Π_node, Π_edge
```

Two main approximations are introduced in order to reduce the computation time, reduce the complexity of the PT system representation, and to account for individuals transportation strategy. We show that the approximations introduced do not affect the results on the overall efficiency of the PT systems.

- We limit the number of total line changes to L_{max} to account for individual choices of not changing line several times. This may lead to overestimate the shortest path lengths for paths with L_{max} changes. In this case, under a locally optimal strategy, one may change line before the best moment. As an example let's consider the following network:
  $(A, B, l1, w = 1)$
  $(B, C, l1, w = 2)$
  $(B, C, l2, w = 1)$
  $(C, D, l2, w = 3)$
  $(C, D, l3, w = 1)$
  The shortest path from A to D with $L_{max} = 1$ is $\{e^{l1}_{A,B}, e^{l1}_{B,C}, e^{l3}_{C,D}\}$ with total weight = 4. However, the algorithm will find the path $\{e^{l1}_{A,B}, e^{l2}_{B,C}, e^{l2}_{C,D}\}$ with total weight = 5.
- The representation includes labelled edges but not labelled nodes, to compromise between a complex description of the system and an efficient one. One way to resolve this issue would be to introduce transfers as links between labelled nodes, which would dramatically increase the network size. Instead, the algorithm includes the transferring time by adding its value to edge weights. The algorithm may overestimate the shortest path lengths in cases were the local optimal strategy of choosing the fastest transportation mean does not provide a globally optimal solution due to long transfer times. However, it provides a very good approximation when the transfer weights are small in comparison with edges weights (i.e. for long distances).

To quantify the impact of the approximations introduced, we calculate shortest paths for the 4 cities considered using both the approximated and the traditional version of the Dijkstra algorithm, where $L_{max} = \infty$, and both nodes and edges are labelled, resulting in a much larger network. For all pairs of nodes (s, t), such that a path with less than L_{max} changes exist, we compute the ratio $r(s, t) = dist_{correct}(s, t)/dist_{approx}(s, t)$, where $dist_{correct}(s, t)$ and $dist_{approx}(s, t)$ are the lengths of the shortest paths computed with the traditional and the approximated versions of the algorithm, respectively. We show the probability density distribution of $r(s, t)$ in figure S2. We find that in all cities the 95% of all paths have the same length in the two cases (see figure S2).

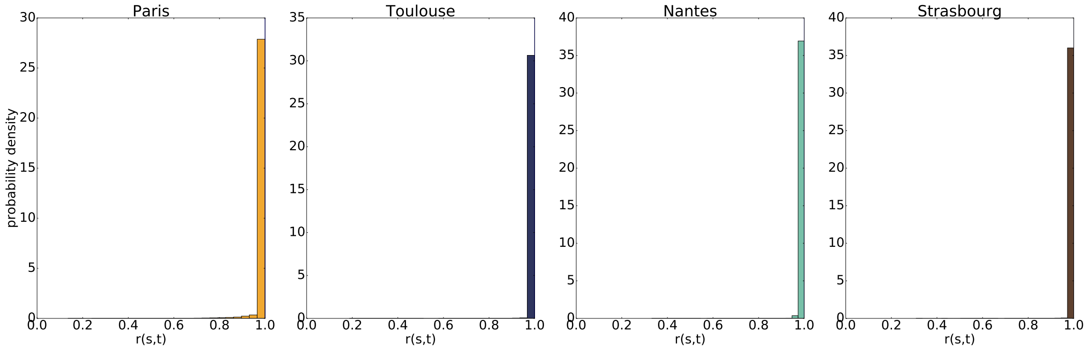

Figure S2. Comparison between the approximated and the traditional Dijkstra algorithm. For each of the cities considered, we show the probability density of paths between nodes s and t with respect to the ratio $r(s, t)$. The 5% percentile is equal to one for all cities.

S4 Pattern detection for Strasbourg, Nantes, and Toulouse

For the urban agglomerations of Strasbourg, Nantes, and Toulouse we detected structural patterns by considering intervals for distances with esolution of $d_2 - d_1 = 5$ kilometers. An example of structure detected for each city is shown in Fig.S3. For an interval range $(d_1, d_2) = (5, 10)km$, both for Strasbourg and Nantes, we observed that the BiCross validation error computed for the adjacency matrix $X_{SP}(5Km, 10Km)$ is similar to the BiCross validation error of the associated random matrix $X_{SPrandom}(0Km, 5Km)$ (Fig.S4). This suggests that there is a lack of structure in the subgraph $G_{SP}(5Km, 10Km)$.

S5 Comparison of the patterns detected and the commuter flows

We further investigate commuters behaviour, by identifying each pair of municipalities such that a flow of commuters exists between them and computing the corresponding PT-car flow ratio as the fraction of commuters using PT over the total people commuting between the two cities. We then compare the cases where the two municipalities are well (Figure S5, A,C,E,G) or badly (Figure S5, B,D,F,H) connected by PT according to our definition, considering the distribution of the PT-car flow ratio.
More precisely, we consider the PT structural pattern network $G_C = (V_C, E_C)$, and the commuter flow network $G^{TM}_{com} = (V^M_{com}, E^{TM}_{com}, W^M_{com})$, where $M = car$ or $M = PT$; first for each edge $(u, v) \in E_C$, we compute the fraction of commuters using PT, $f(u, v) = (W^{PT}_{com}(u, v) + W^{PT}_{com}(v, u))/(W^{PT}_{com}(u, v) + W^{PT}_{com}(v, u) + W^{car}_{com}(v, u) + W^{car}_{com}(v, u))$.Then, we compute the same quantity for all edges $(u, v) \in E_{com}$ that are not in E_C. For each city, we finally look at the distribution of $f(u, v)$ for both well and badly connected municipalities (Figure S5).

In the case of Paris agglomeration, there is a significant difference between the case of privileged connections, where the distribution is left-side skewed (figure S5 A), and not privileged connections,

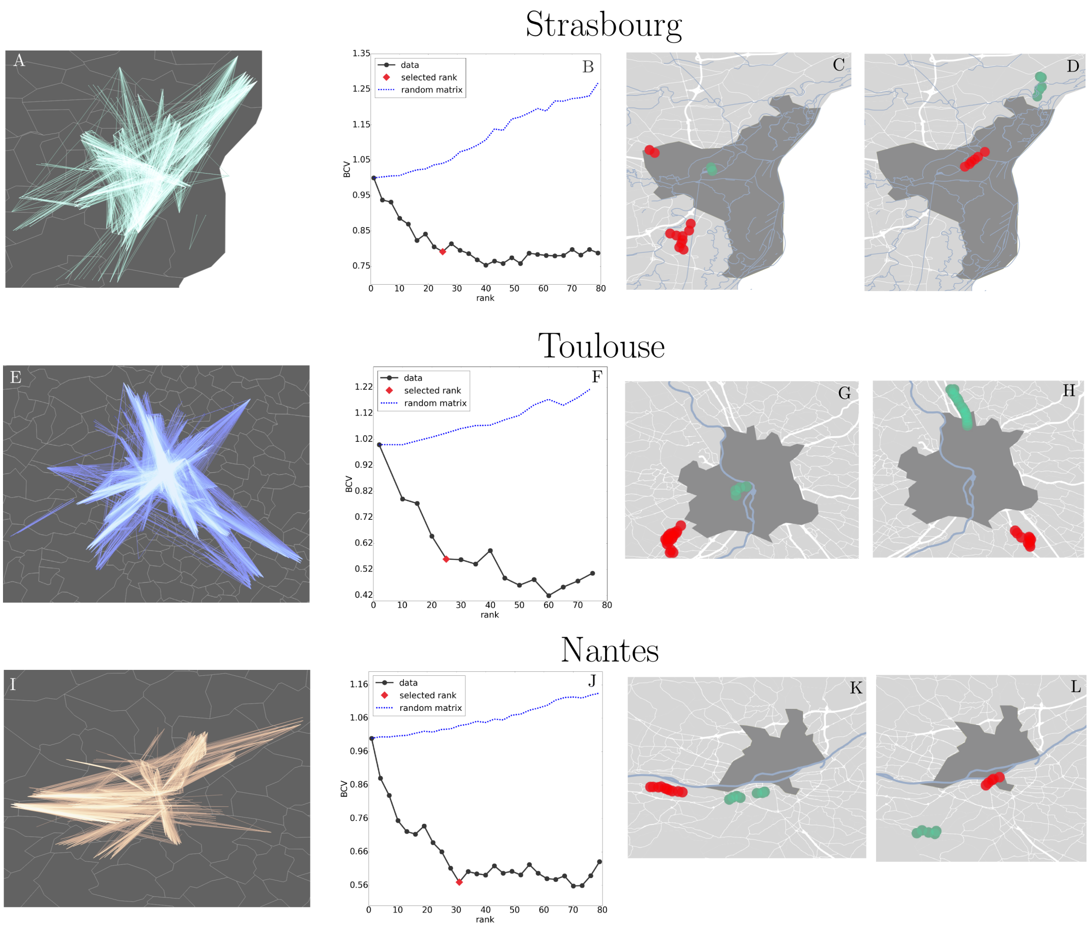

Figure S3. Pattern detection using the multi-edge P-space representation. For Strasbourg, Toulouse, and Nantes, we show respectively in A,E and I the geographic representation of graph G_{SP}, where links correspond to the 1% best shortest paths of the public transportation network. In B, F and J, we show the normalised BiCross validation errors computed for the adjacency matrix $X_{SP}(0Km, 5Km)$ (grey full line) of the same graphs, for the associated random matrix $X_{SPrandom}(0Km, 5Km)$ (dashed line). The selected number of structures k_s is marked with a red rhombus. In C and D,G and H,K and L, two examples of structures revealed in the PT system are presented. Green dots are in-going, while red dots are out-going affiliated.

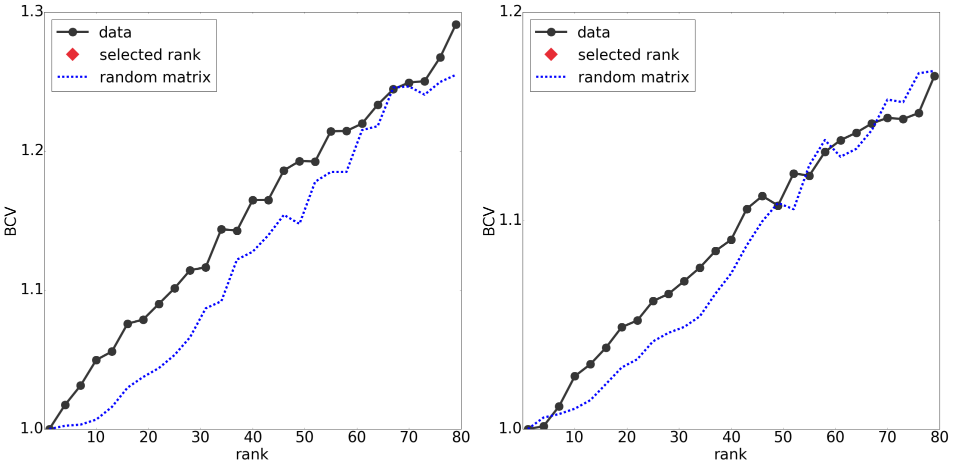

(A) Strasbourg,$d = 5 - 10Km$ **(B)** Nantes,$d = 5 - 10Km$

Figure S4. For the cities of Strasbourg (a), and Nantes (b), the normalised BiCross validation error computed for the adjacency matri $X_{SP}(5Km, 10Km)$ (grey full line) is similar to the BiCross validation error of the associated random matrix $X_{SPrandom}(0Km, 5Km)$ (dashed line). Therefore, no rank is selected and structures were not extracted.

where the distribution is more symmetrical (figure S5 A). This indicates that when the PT provides with good transportation according to our method, commuters prefer PT with respect to car. On the other hand, for Toulouse, Nantes and Strasbourg agglomerations, there is significantly less difference in the distribution of the PT-car flow ratios for well and badly connected pairs of cities. On the one hand, this may suggest commuters tend to use the car even where good connections are provided. On the other hand, we have to consider both that our selection was less strict for these cities, and that self loops (inter-city connections) may play an important contribution which could not be considered here due the resolution limit of the commuter dataset.

S6 Characteristics of privileged connections

In this section we show some of the characteristics of the selected privileged connections. For each city, we show the distribution of the number of line changes (Figure S6), of the number of different modalities in the same path (Figure S7), and the occurrences of each possible transportation modality (Figure S8), as a function of the shortest path distance.

S7 Comparison with the single-layer representations

The straightforward graph representation S9, widely used for PT systems, where for each modality stops correspond to PT stops, and edges connect consecutive stops (connected by a vehicle without stopping between stops) does not allow to identify privileged connections and well-connected areas within the city.
In the MS (MS, figure 4A) we showed the city profile obtained considering privileged connections in the multilayer representation. Here, we compare the city profile with the one obtained considering the transportation modalities separately. We compute the shortest paths taking into account only buses, metro and rail connections, and we select privileged connections in the same way detailed for the entire multi-layer (see MS, section 3a). We show on the same figure (Figure S10, left) the profiles obtained for each transportation modality in Paris. The city single-layer profile differs from the one obtained considering all transportation modalities (Figure S10, right) since the advantages due to the interconnectedness of several transportation modes are not accounted.

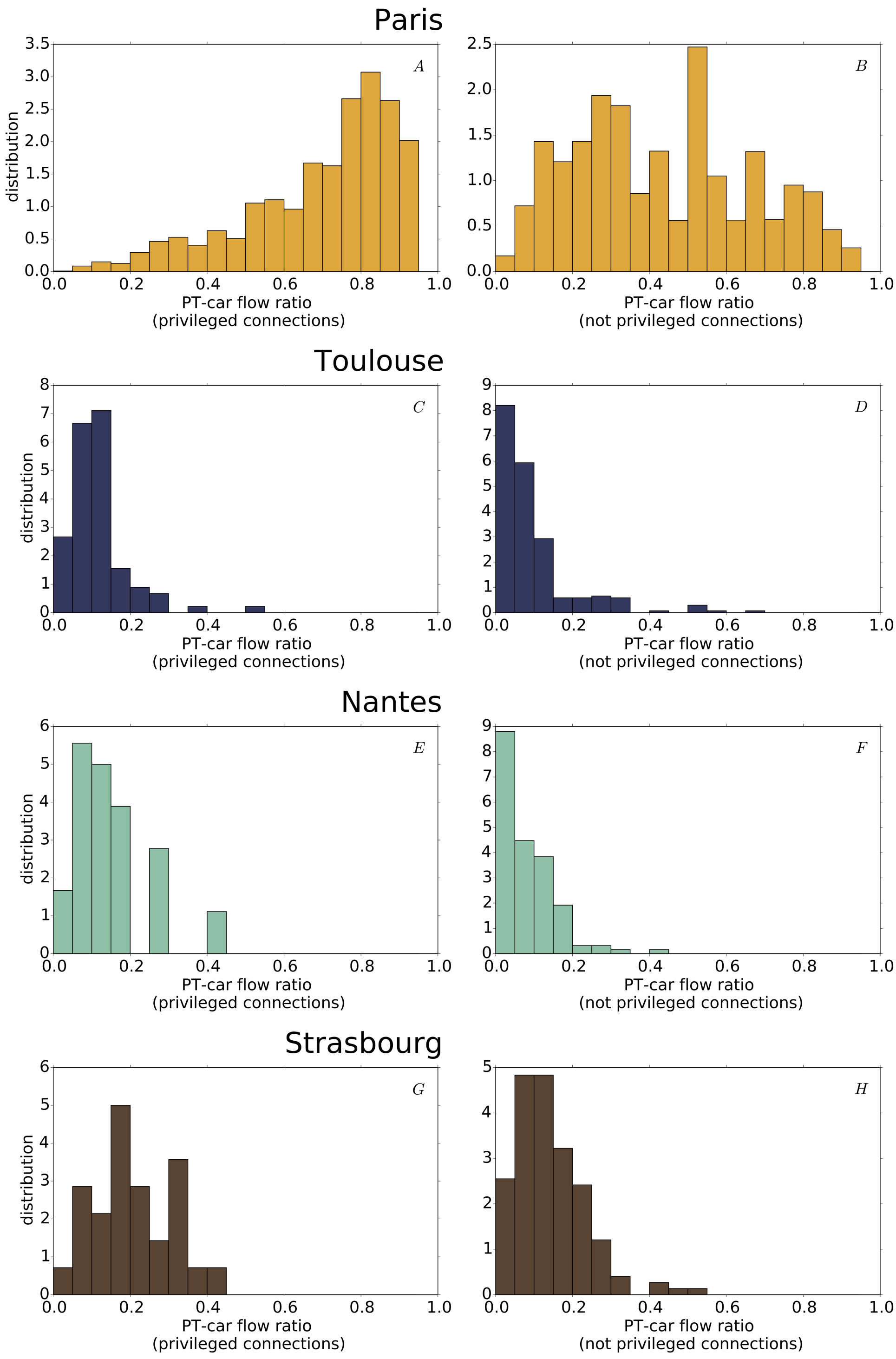

Figure S5. For each city, we show the distribution of the PT-car flow ratio $f(u,v)$ when u and v are well (as defined in the main text) connected (A) or badly (the complementary connections) connected (B)

.

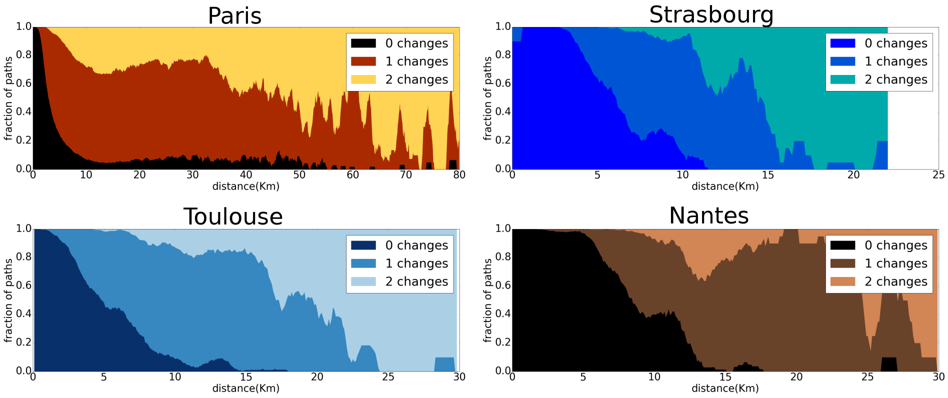

Figure S6. We show for each city the fraction of privileged shortest paths with 0, 1, or 2 number of line changes in different colors, as a function of the shortest path distance.

.

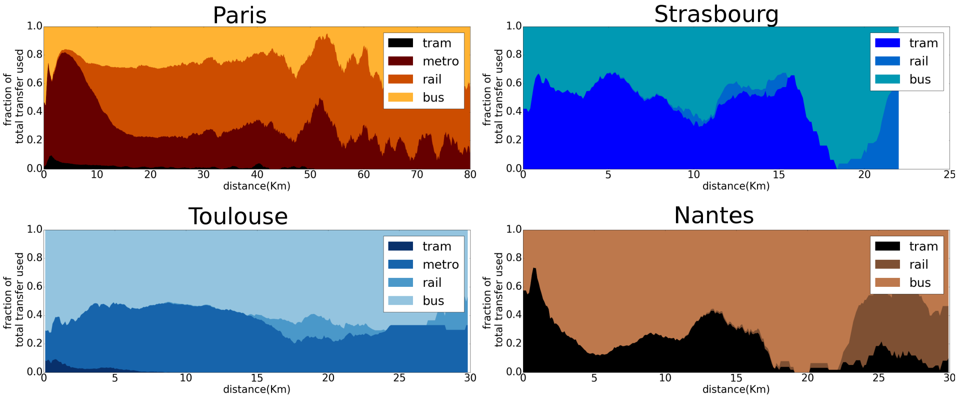

Figure S7. For each city, we consider all edges occurring in privileged shortest paths. As a function of the shortest path distance, we show the fraction of edges according to their transportation modality.

.

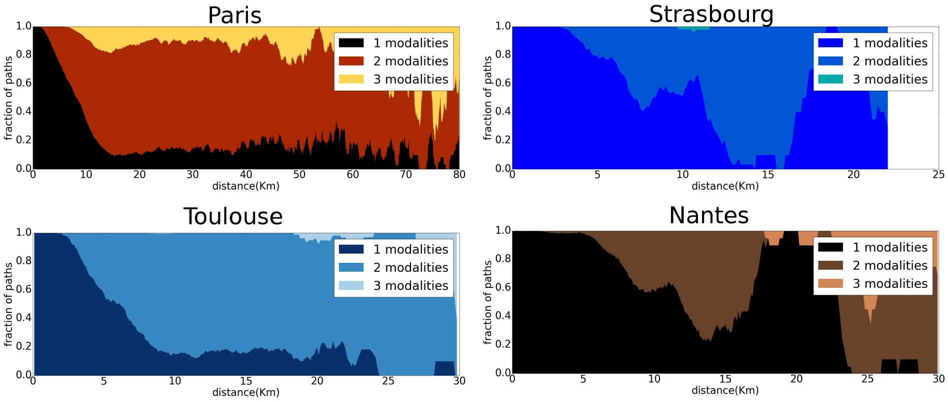

Figure S8. We show for each city the fraction of privileged shortest paths including 1, 2, or 3 transportation modalities in different colors, as a function of the shortest path distance.

.

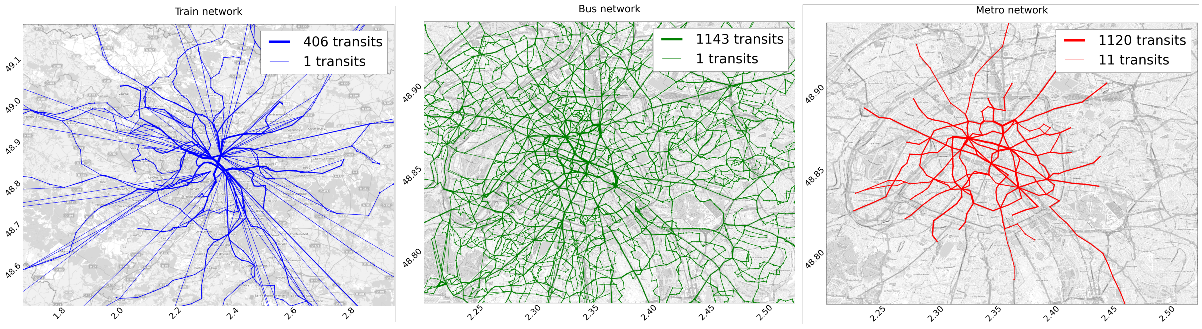

Figure S9. Naive representation of the multi-layer PT system of Paris. For each modality (Tram, Bus, Metro, from left to right), dots correspond to nodes, and connecting edges have a thickness proportional to edge the number of transits per day

.

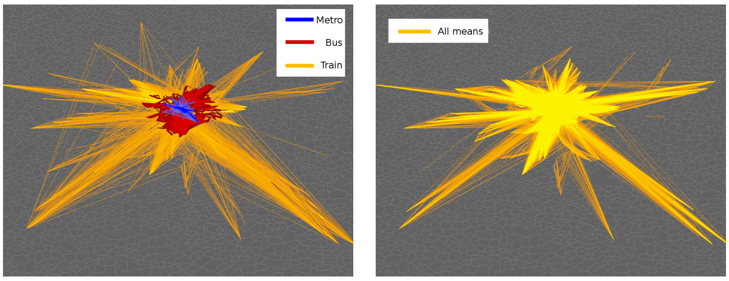

Figure S10. Comparison between the city profile of Paris considering the single-modality single-layer representation (left), and the multi-layer representation (right).

.

References

[1] General transit feed specification reference. 2012 `https://goo.gl/W6M1B3`

[2] Enquête nationale transports et déplacements. 2007-2008 `http://goo.gl/0Vlq8H`

[3] Fichier mobilités professionnelles des individus: déplacements commune de résidence / commune de travail. 2010 `http://goo.gl/SM2YCU`

[4] Google maps apis. 2015 `https://goo.gl/Gzujaf`

[5] Base communale des aires urbaines. 2010 `http://goo.gl/HXc1g0`

[6] Lee, D. D. & Seung, H. S. 2001 Algorithms for non-negative matrix factorization. in *Advances in neural information processing systems* (MIT Media Press), 556–562.

[7] Brunet, J.-P., Tamayo, P., Golub, T. R. & Mesirov, J. P. 2004 Metagenes and molecular pattern discovery using matrix factorization. *Proceedings of the national academy of sciences* **101**, 4164–4169.
